# A beta version of life: p110β takes center stage

**DOI:** 10.18632/oncotarget.207

**Published:** 2010-12-30

**Authors:** Hashem A. Dbouk, Jonathan M. Backer

**Affiliations:** Department of Molecular Pharmacology, Albert Einstein College of Medicine, Bronx, NY, USA

**Keywords:** PIK3CB, PTEN, lipid kinase, oncogenic transformation

## Abstract

The PI3K pathway is frequently activated in tumors, most commonly through p110α mutation or PTEN deletion. In contrast to p110α, p110β is oncogenic when over-expressed in the wild-type state, suggesting that its regulation by p85 is different than that of p110α. In this perspective, we summarize recent data concerning the regulation of p110β, which shows that wild-type p110β acts like an oncogenic mutant of p110α. We also discuss the significance of this altered regulation in tumor models of PTEN deletion, as well as the potential implications of the unique p110β regulation on GPCR-driven tumorigenesis.

Phosphoinositide 3-kinases (PI3Ks) are a family of enzymes that catalyze the phosphorylation of the D3 hydroxyl of the inositol ring in phosphoinositides. The three classes of PI3Ks are distinguished by sequence homology and substrate specificity *in vivo* [1]. Class I PI3Ks signal downstream from Receptor Tyrosine Kinases (RTKs) and G-protein coupled receptors (GPCRs) and phosphorylate PI(4,5)P2 to generate PI(3,4,5)P3 (PIP3), an important second messenger that recruits proteins containing a PH domain [2]. Class IA PI3Ks are obligate heterodimers of a catalytic subunit (p110α, β, δ) with regulatory subunit (p85α, p85β, p55α, p50α, and p55γ), and Class IB PI3Ks are dimers of a p110γ catalytic subunit and p101 or p87 regulatory subunits. The canonical classification of PI3Ks defines Class IA PI3Ks as signaling downstream from RTKs [3], whereas Class IB PI3Ks signal downstream from GPCRs [1]. This distinction has been questioned by data showing that the p110β isoform of class IA PI3Ks is activated by Gβγ subunits downstream of GPCRs, similar to p110γ [4-7]. A large number of recent studies has defined signaling differences between the p110β and the p110α catalytic subunits (reviewed in [8]).

Class I PI3K signaling is frequently amplified in tumors, most commonly by activating mutations in PIK3CA (which codes for p110α) and disabling mutation or deletion of PTEN (phosphatase and tensin homolog) [9]. In contrast to p110α, no oncogenic mutations have been found in any of the other class I PI3K catalytic subunits. However, p110α is only oncogenic when mutated, whereas p110-β, -γ, and -δ are oncogenic when expressed in their wild-type form [10]. This suggests that the regulation of p110β and p110δ is different than that of p110α. Recent studies have shown that p110β but not p110α has essential roles in tumorigenesis in PTEN-null mouse models and cell lines [11, 12]. p110β has also been implicated in the growth of ErbB2-driven mammary tumors [13] and in Ras-driven tumors [12]. Thus, defining the mechanism of p110β regulation could have important clinical implications.

We have previously shown that C2-iSH2 contacts formed by N345 of p110α with D560/N564 in p85 are required for full inhibition of p110α activity by p85. These contacts are disrupted by an N345K mutation in p110 and by point mutants (p85D560K/N564K) or truncations (p85-572^STOP^) in p85 [14, 15]. Furthermore, we described an assay to measure the presence or loss of the C2-iSH2 interface. Wild-type p110α is strongly inhibited by p85 but minimally inhibited by p85D560K/N564K or p85-572^STOP^. In contrast, p110α-N345K shows the same minimal inhibition by wild type p85 or the p85D560K/N564K and p85-572^STOP^ mutants. Therefore, the differential regulation of p110 molecules by wild-type versus mutant p85 can be used to detect the presence of an intact C2-iSH2 interface.

We have now used this assay to study the regulation of p110β as compared to the other class IA PI3K catalytic subunits, p110α and p110δ [16]. Sequence alignment of p110β with p110α shows a crucial difference in the C2 domain of p110β, with K342 of p110β aligned with N345 of p110α. This makes wild-type p110β analogous to the oncogenic p110α mutant N345K. Using our assay for the C2-iSH2 interface, we showed that p110β is minimally inhibited by wild-type p85 or p85 572^STOP^, similar to the p110α N345K mutant. A mutant p110β-K342N that mimics the C2-iSH2 interface in p110α is less transforming than wild-type p110β, and shows a gain-of-function for differential regulation by wild-type p85 versus p85-572^STOP^. p110β-K342N is still regulated by Gβγ subunits, similar to wild-type p110β.

Further analysis of the role of the C2-iSH2 interface in the transforming potential of p110β was performed using p110α/β chimeras. Chimeric p110α/β molecules having the C2 of p110α showed decreased transforming potential as compared to p110β, whereas a p110α/β chimera containing the C2 domain of p110β shows the high transforming potential characteristic of p110β. Our data show that the transforming potential of p110β is due, at least in part, to the disruption of the inhibitory interface between the C2 of p110β and the iSH2 domain of p85, which leads to high basal p110β signaling [16]. In contrast to p110β, p110δ showed the differential regulation by wild type versus mutant p85 that is characteristic of an intact C2-iSH2 interface. Given that p110δ is also transforming in its wild type state, its enhanced transforming potential must be due to other factors [16].

In addition to the impact of a disrupted C2-iSH2 interface on the transforming potential of p110β, the loss of p85 inhibition might explain the inability of p110β to signal downstream of receptor tyrosine kinases [7]. Activation of class IA PI3Ks by phosphopeptides involves the disruption of an inhibitory contact between the nSH2 domain of p85 and the helical domain of p110 [17]. If p110β is less inhibited by p85 under basal conditions, this would lead to a loss of activation of p85/p110β dimers by activated RTKs. This is supported by recent data showing that cancer specific p85 mutations in the nSH2 and iSH2 domains function solely through p110α, not p110β [18].

Several studies have suggested that p110β is the sole class IA PI3K catalytic subunit required for initiation and maintenance of PTEN-null driven tumors [11, 12, 19]. It is also interesting that PTEN seems to specifically associate with p85/p110β, an interaction mediated by the SH3 and BH domains of p85 and leading to enhanced PTEN catalytic activity [20, 21]. The association of p110β with PTEN is consistent with our data showing that purified p110β is relatively active under basal conditions [16], as it provides a regulatory mechanism to prevent uncontrolled signaling in normal cells. Thus, under normal growth conditions, the activity of p110β is antagonized by the PTEN associated with the p85/p110β dimer, thereby controlling steady-state PIP3 levels. However, in conditions where PTEN expression or activity is lost, the activity of p110β is no longer countered by the phosphatase activity of PTEN, leading to high levels of PIP3 and downstream signaling. This may provide a model for the deregulation of PI3K signaling and p110β-dependence of PTEN-null tumors (Figure 1). It is also interesting to note that PTEN has been shown to function downstream of anti-migratory GPCRs [22] and to negatively regulate CXCR4-mediated chemotaxis [23]. This suggests that PTEN might also be acting as a negative regulator of p110β activation downstream of GPCRs involved in the regulation of cell motility.

**Figure 1:**
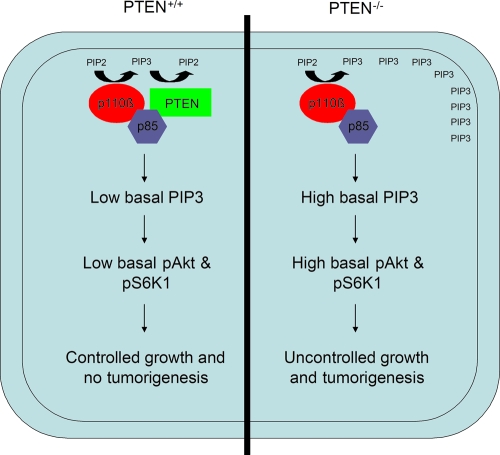
Model for p110β dependency in PTEN null tumors p85 binds to both p110β and PTEN, allowing negative regulation of basal p110β activity by PTEN. This ensures that normal (PTEN^+/+^) cells maintain a low basal level of PIP3 at the plasma membrane, leading to low basal signaling and controlled cell growth. In contrast, in PTEN^−/−^ cells, basal p110β activity is not countered by p85/p110β-associated PTEN, leading to high basal PIP3 levels in the membrane, enhanced activation of Akt and S6K1, and uncontrolled cell growth and tumorigenesis.

p110β is essential for survival and has unique functions that are not redundant with other class IA PI3K catalytic subunits, as knockout mice show embryonic lethality [24]. p110β is the sole class IA PI3K subunit that signals downstream of GPCRs via direct Gβγ binding and activation, and the only GPCR-regulated PI3K in non-hematopoietic cells. The mechanism of Gβγ-mediated regulation of p110β is not well characterized. Previous studies with p110γ have shown that Gβγ binds to N-terminal and C-terminal regions of p110γ and activates the kinase activity of p110γ [25]. Gβγ also binds to p101 to mediate membrane recruitment of the p101/p110γ complex [26]. For p110β, activation requires direct interaction of Gβγ subunits with the p110β catalytic subunit and appears to be independent of the p85 regulatory subunit [27]. Using chimeric p110α/β molecules, we have narrowed the interaction interface with Gβγ to the helical-kinase domains of p110β [16]. Further delineation of this interacting interface will be important for targeting the subset of p110β functions that are downstream of GPCRs. Mutations in the Gβγ binding site of p110β will be important for defining the role of p110β in initiating GPCR-driven tumors, and for studying its contribution to invasion and metastasis triggered by GPCR ligands.

In addition to transmitting signals downstream of GPCRs, p110β has been shown to be essential for clathrin-mediated endocytosis [12, 13] and autophagy [28]. These roles are suggested to be mediated by interactions with Rab5, and are unique in that they are kinase-independent functions of p110β. Furthermore, p110β regulates integrin mediated signaling in platelets [29, 30], and may have important antithrombotic roles [31]. This is intriguing because integrin signaling and integrin and focal adhesion endocytosis, which is mediated by clathrin [32], are essential for cell migration [33]. It will be important to determine whether the role of p110β in endocytosis is related to its functions in cancer cell migration and invasion.

p110β is unique among the class IA PI3Ks, both in terms of functions and regulation. The isoform-specific regulation of p110β by Gβγ and Rab5, as well as its critical roles in a subset of tumor types, may lead to novel therapeutic approaches for the treatment of human cancer.
